# Immersive technologies for tourism: a systematic review

**DOI:** 10.1007/s40558-022-00228-7

**Published:** 2022-06-22

**Authors:** Eko Harry Pratisto, Nik Thompson, Vidyasagar Potdar

**Affiliations:** 1 Discipline of Business Information Systems, School of Management and Marketing, Curtin University, Perth, WA Australia; 2 Department of Informatics Engineering, School of Vocational, Sebelas Maret University, Surakarta, Indonesia

**Keywords:** Augmented reality, Immersive technology, Mixed reality, Systematic review, Tourism, Virtual reality

## Abstract

This review provides a comprehensive view of immersive technology in tourism by critically analysing prior scholarly work. More specifically, it identifies the recent use of immersive technology in this field and the potential challenges it poses. This systematic review follows PRISMA guidelines and involves four key steps—identifying research questions, defining keywords, selecting studies based on inclusion and exclusion criteria, and synthesising results. It focuses on immersive technology in tourism-related peer-reviewed journal articles published from 2012 to 2020. The papers were selected from ten prominent journal databases. Some databases used combinations of search queries but with inclusion and exclusion criteria. This systematic review builds on the existing reviews by adding knowledge regarding state-of-the-art immersive technology usage in tourism and its integration with other technology. This review additionally identifies the potential challenges of implementing immersive technology in tourism. Finally, it presents a set of directions for future research in this space. In practice, the findings from this review can make both software developers and tourism providers aware of the potential of immersive technology in tourism. Software developers might consider appropriate designs that suit such usage, and tourism providers might consider using immersive technology to promote tourism destinations and provide a support system to maximise the benefits of immersive technology.

## Introduction

Tourism represents a product of modern, complex society (Walton 2018) and is traditionally defined as people travelling to a destination outside of their usual home and work environments for leisure (United Nations World Tourism Organization 2019). The tourism industry has been deeply affected by rapid technological change (tom Dieck et al. 2018c), which has been felt even before the restrictions on personal movement caused by the global COVID-19 pandemic. Technology can offer new experiences in a simulated environment (e.g. immersive technology) without requiring physical travel. Immersive technology thus provides a suitable environment for tourism promotion, experience enhancement, or education (Guttentag 2010; Bekele et al. 2018). Advances in foundational technology now blur the boundary between the real world and the virtual environment by giving users an experience with a sense of immersion (Lee et al. 2013a, b). From this perspective, immersive technology enables tourism stakeholders to enhance tourists’ satisfaction since consumers can choose and modify such an experience to a degree that was once considered impossible (Williams and Hobson 1995).

**Fig. 1:**
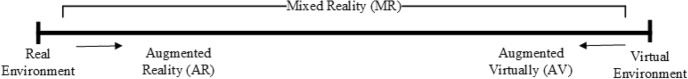
Reality–virtuality continuum (Milgram et al. 1995)

Immersive technology concepts can be considered on a reality-virtuality continuum (see Fig. 1): at the former end is a real environment, and at the latter end is a computer-generated virtual environment. Within that spectrum are two concepts—augmented reality (AR) and augmented virtuality (AV), which fall under the umbrella terminology of mixed reality (MR). In addition, there is virtual reality (VR), which is a fully virtual environment.

In the context of this study, immersive technology blurs the boundary between the real physical world and the virtual world, letting users experience a sense of immersion (Slater and Wilbur 1997). Referring to the reality–virtuality continuum, as the direction from the physical world point moves toward the virtual environment end, the technology delivers more virtual elements on the device’s screen. This means that the number of virtual objects seen by users increase, whereas the number of physical objects they see decreases. Most notably, immersive technology includes AR and VR. Whereas AR can overlay the view of the user’s current environment with digital objects (Azuma 1997), VR can create a virtual environment that the user can seamlessly interact with in real time (Guttentag 2010). Both AR and VR can increase the quality of visitors’ experience of a destination (Yung and Khoo-Lattimore 2019).

The VR industry is proliferating, with a projected increase in market size from 6.2 billion US dollars in 2019 to more than 16 billion US dollars in 2022 (Alsop 2020). This is in stark contrast with the tourism industry. The United Nations World Tourism Organization (2019) reported approximately 180 million fewer international arrivals between January and March 2021 than in the first quarter of 2020. Expressly, the number of international tourist arrivals worldwide in 2020 declined by 73% compared to 2019, and by another 83% in 2021 compared to 2020. Immersive technology is still viable even though the tourism industry in many regions was put on hold in 2020 due to COVID-19 travel restrictions. For example, in Australia, the number of visitor arrivals declined in February 2020 when the Australian Government first introduced travel restrictions. International arrivals fell 99.6% compared to the previous year (Tourism Australia 2020). As the global pandemic continues, a higher potential exists for immersive technology to become a viable alternative to travelling.

Immersive technology has been adopted and implemented in various tourism areas. The technology provides a surrogate experience that can be used to convince potential visitors to travel to a tourism destination (Chung et al. 2018; Flavián et al. 2019; Lee et al. 2019; Kim et al. 2020). The benefit usage of immersive technology includes navigation systems (Balduini et al. 2014; Sommerauer and Müller 2014), tourism promotion (Lacka 2020; Li and Chen 2019; Kim et al. 2020), and enhanced user experiences during visitation (Puig et al. 2020; Errichiello et al. 2019; Rodrigues et al. 2019).

Researchers such as Baker et al. (2017), Beck et al. (2019), Wei (2019); and Yung and Khoo-Lattimore (2019) have conducted reviews on AR and VR in tourism. Wei (2019) located major key dimensions of user behaviour in prior AR and VR-related studies using a framework representing stimuli, decisions and consequences cause-and-effect relationships. The author also discussed the development of theory and methodology within AR and VR in tourism research. In addition, Yung and Khoo-Lattimore (2019) reviewed AR and VR usage in the tourism sub-sectors of marketing, education, and tourism experience enhancement. These reviews are valuable for understanding immersive technology adoption in tourism concerning their specific areas of interest. However, questions remain regarding the extent of immersive technology usage in tourism and its potential challenges. Identifying and mapping the recent immersive technology development in tourism will help researchers identify the technology usage trends and determine the important areas for further investigation.

To explore this timely area of technological development and research, we present in this article a systematic review of the current state of research into immersive technology use in tourism. Therefore, this review aims to build knowledge on what has been investigated about immersive technology in tourism from existing literature. Additionally, this review includes suggestions for future research. The systematic review is achieved through the following objectives: (1) extract related existing literature from databases from a specified period, (2) select the literature based on inclusion criteria, (3) synthesise the selected literature to answer the research questions, and (4) identify research gaps for future research recommendations.

## Existing reviews

This review identified four prior review articles (see Table 1). One AR-related review (Baker et al. 2017) focused on mobile AR for hard-of-hearing visitors. Beck et al. (2019) focused on VR, classifying it based on the immersive level. Two reviews (Yung and Khoo-Lattimore 2019; Wei 2019) addressed how both AR and VR are used in the tourism context in general. This section discusses each of the previous reviews’ scope to highlight their differences.

**Table 1:** Summary of prior review articles on immersive technology in tourism

References	Scope	Protocol	Databases/type of literature included	Keywords	No. of articles	Timespan	Key findings
Baker et al. (2017)	Mobile AR applications for deaf and hard of hearing visitors	Systematic literature review Expert opinion	IEEE, SpringerLink, World Scientific, and ScienceDirect AR-related to hearing impaired	Mobile augmented reality engagement	11	Not mentioned	Eleven elements to trigger engagement with mobile augmented reality for hard of hearing visitors at museums and galleries
Beck et al. (2019)	A comprehensive review of VR and its classification based on the immersive level (non-immersive, Semi-immersive, Full-immersive)	State-of-the-art review	ScienceDirect and Google Scholar Peer-reviewed conference proceedings and journal articles	VR tourism, VR technology in tourism, immersive tourism, 360-degree tourism, virtual tourism, and virtual environment (VE) in tourism	27	2000–2018	VR classification based on the immersive level including its definition VR challenges in tourism Potential use of VR during pre-travel phase and on-site
Wei (2019)	AR and VR in tourism and hospitality	Literature review	Sage, ScienceDirect, Emerald, EBSCOhost Peer-reviewed journal articles	Virtual reality, augmented reality, hospitality, tourism	60	2000–2018	Key constructs within a framework (stimuli, dimensions, consequences) on VR/AR application in tourism and hospitality Theoretical development in AR and VR studies Research methodologies development on AR and VR studies
Yung and Khoo-Lattimore (2019)	AR and VR application in tourism sector	Systematic quantitative review	Scopus, EBSCO, Elsevier, ProQuest, and Emerald Peer-reviewed journal articles	‘Augmented realit*’, ‘virtual realit*’, ‘virtual world*’, ‘virtual environ*’	46	Any studies until 2016	AR and VR in marketing, education, experience enhancement, food and beverage, and Meetings, Incentives, Conventions, and Exhibitions (MICE) Theory-based VR and AR tourism research

All the existing reviews had similar methodologies, including searching for articles in selected databases, screening the articles using inclusion and exclusion criteria, and reporting findings. ScienceDirect was the most used database in three reviews (Baker et al. 2017; Beck et al. 2019; Wei 2019), followed by Emerald and EBSCOhost. The studies by Wei (2019) and Yung and Khoo-Lattimore (2019) only included peer-reviewed journal articles, in contrast with Beck et al. (2019), who also included peer-reviewed conference papers. Baker et al. (2017) did not state which type of articles were included.

The existing reviews revealed interesting findings regarding immersive technology implementation in tourism. For example, Baker et al. (2017) identified 11 major elements required to provide a mobile AR system for hard-of-hearing visitors. Those elements might be useful to ensure that the targeted user receives the correct information from the AR system. Two other studies were concerned with the terminology surrounding the technology. Yung and Khoo-Lattimore (2019) highlighted AR and VR-related terminology issues: several terms (virtual environment, VR and virtual world) were used inconsistently.

Similarly, Beck et al. (2019) focused on VR classification, including non-immersive, semi-immersive, and fully immersive VR in tourism. The authors argued that VR should deliver high-quality images to help users avoid motion sickness and encourage them to visit the destination in real life. Wei (2019) examined AR and VR research development in hospitality and tourism. The author identified major dimensions and classified them using the stimuli–dimension–consequence framework.

Some suggestions for future research can be derived from the existing reviews. A study is needed that focuses on technical aspects such as content, design, interactivity (Beck et al. 2019) and cross-cultural approaches (Wei 2019) to understand how users perceptions of immersive technology might vary. A comparison study could also examine the usage of immersive technology such as AR, VR and MR in tourism. Finally, Yung and Khoo-Lattimore (2019) suggested that future research identify the impact of having AR or VR booths in travel agencies and information centres and the possible applications of VR images or videos produced from 360° cameras.

Based on the existing reviews’ scopes, we identified the distinct new contributions made in our work. First, this review complements the findings on VR and AR presented by Wei (2019) and Yung and Khoo-Lattimore (2019) and the use of this technology in tourism sectors, including VR with 360° technology. Second, this study covers all immersive technology applications in tourism research rather than focusing only on AR (Baker et al. 2017) or VR (Beck et al. 2019). Finally, this review considers the characteristics of immersive technology, its integration with other technology and potential challenges.

## Methodology

This study utilises a systematic literature review to answer three research questions related to immersive technology in tourism by summarising research findings to obtain a comprehensive view of the state-of-the-art use of immersive technology and identify potential issues for future research. This section details the systematic literature review process by implementing a guideline proposed by Okoli (2015).

### Identifying the research questions

In the previous section, we distinguished this review’s contribution from that of previous review articles. This review focuses on state-of-the-art immersive technology in tourism to answer several research questions. We followed the PICO framework (Pollock and Berge 2018) to develop research questions based on the aim of this review. The research questions are as follows:

- **Research question 1 (RQ1)**: What characteristics of immersive technology are used in tourism research?
- **Research question 2 (RQ2)**: To what extent does immersive technology play a role in the tourism visiting experience?
- **Research question 3 (RQ3):** What are the potential challenges of developing immersive technology for the tourism domain?

### Defining search keywords

Given the objective of this study, keywords needed to be defined to obtain relevant articles from databases. Our article search strategy included all published articles related to AR, VR, and MR since those terms are within the domain of immersive technology. The keywords ‘augmented reality’, ‘virtual reality’, ‘mixed reality’, ‘360 video’, ‘360 panoramic’, and ‘360 degree’ were included since these are present in many VR-related studies. The query also included the keywords ‘tourist’, ‘tourism’, and ‘visitor’ to keep the focus on tourism. The searching technique consisted of combined keywords and Boolean operators such as ‘AND’ and ‘OR’ to narrow the results. We included articles published from 2012 until 2020 to obtain an insight into the use of state-of-the-art immersive technology in tourism. We also only included articles published in peer-reviewed journals in English. Articles from proceedings, conferences, magazines, and books were excluded from this review. The search query was then executed on the following ten electronic databases: ACM Digital Library, EBSCOhost, Emerald Insight, IEEE Xplore, ProQuest, SAGE, ScienceDirect, Taylor and Francis, Web of Science and Scopus, considering the boundaries of the various definitions of immersive technology, time range, keywords, and type of articles. We used ten databases to ensure that we did not miss any relevant articles. Emerald Insight, Web of Science, and Scopus use a slightly different syntax, meaning we changed the search query slightly to suit their characteristics. The search query we developed to guide the literature search is outlined in Table 2. The search query was applied to titles, abstracts and keywords in selected databases.

**Table 2:** The search query for databases

Database	Search query
Emerald Insight	(content-type:article) **AND** (abstract:"augmented reality" OR (abstract:"virtual reality") OR (abstract:"mixed reality") OR (abstract:”AR") OR (abstract:"VR") OR (abstract:"MR") OR (abstract:"360 video") OR (abstract:"360 panoramic") OR (abstract:"360 degree")) **AND** (abstract:"touris*" OR (abstract:"visit*"))
Scopus	TITLE-ABS-KEY ("virtual reality" OR "augmented reality" OR "mixed reality" OR “AR” OR “VR” OR “MR” OR "360 video" OR "360 panoramic" OR "360 degree") **AND** TITLE-ABS-KEY (touris* OR visit*) **AND** (LIMIT-TO (PUBSTAGE, "final")) **AND** (LIMIT-TO (DOCTYPE, "ar") OR LIMIT-TO (DOCTYPE, "re")) **AND** (LIMIT-TO (LANGUAGE, "English")) **AND** PUBYEAR > 2011 AND PUBYEAR < 2021
Web of Science	TS = ("virtual reality" OR "augmented reality" OR "mixed reality" OR “AR” OR “VR” OR “MR” "360 video" OR "360 panoramic" OR "360 degree") **AND** TS = (touris* OR visit*)
Other databases	("virtual reality" OR "augmented reality" OR "mixed reality" OR “AR” OR “VR” OR “MR” OR "360 video" OR "360 panoramic" OR "360 degree") **AND** (touris* OR visit*)

### Study selection

As part of the study selection stage, inclusion and exclusion criteria were defined to produce relevant articles to the research questions. This review excluded inappropriate terms, such as ‘non-immersive VR’, often applied during the article evaluation process. For example, we only included articles using applications with a first-person perspective. Articles using applications with a third-person perspective, such as Second Life (Linden Research 2019), were excluded. Articles discussing VR technology and covering almost all of the user’s range of vision through, for example, image or video projection on the surrounding walls (Ghadban et al. 2013) were included in this study.

Regarding the VR content, 360° images and video are common types of content found in the selected articles. Such content is preferable for promoting tourism destinations because it gives the potential tourist a view of the prospective destination most like real life. The computer-generated virtual environment might be suitable for reconstructing a specific situation or learning context.

Table 3 details a full list of inclusion and exclusion criteria for the screening process of the selected articles. The search query generated 1017 articles from the ten databases (see Table 4).

**Table 3:** Inclusion and exclusion criteria for study selection

	Criteria
Inclusion	Articles published from 2012 to 2020 Journal article Full-text article Peer-reviewed Empirical (qualitative, quantitative, mixed-methods, design science) and conceptual articles related to the use of AR or VR or MR in tourism VR using 360-video or 360 images
Exclusion	Papers written in a language other than English Articles related to reconstruction or software/hardware optimisation Third-person point of view of non-immersive VR application Article from proceedings, conferences, magazines, and books

**Table 4:** Search results from ten databases

Database	Number of articles
ACM Digital Library (https://dl.acm.org/)	25
EBSCOhost (https://search.ebscohost.com/)	35
Emerald (https://www.emerald.com/insight/)	56
IEEE Xplore (https://ieeexplore.ieee.org/)	11
ProQuest (https://search.proquest.com/)	51
Sage (https://journals.sagepub.com/)	23
ScienceDirect (https://www.sciencedirect.com/)	74
Scopus (https://www.scopus.com/)	432
Taylor and Francis (https://www.tandfonline.com/)	27
Web of Science (https://www.webofknowledge.com/)	283
Total	1,017

**Fig. 2:**
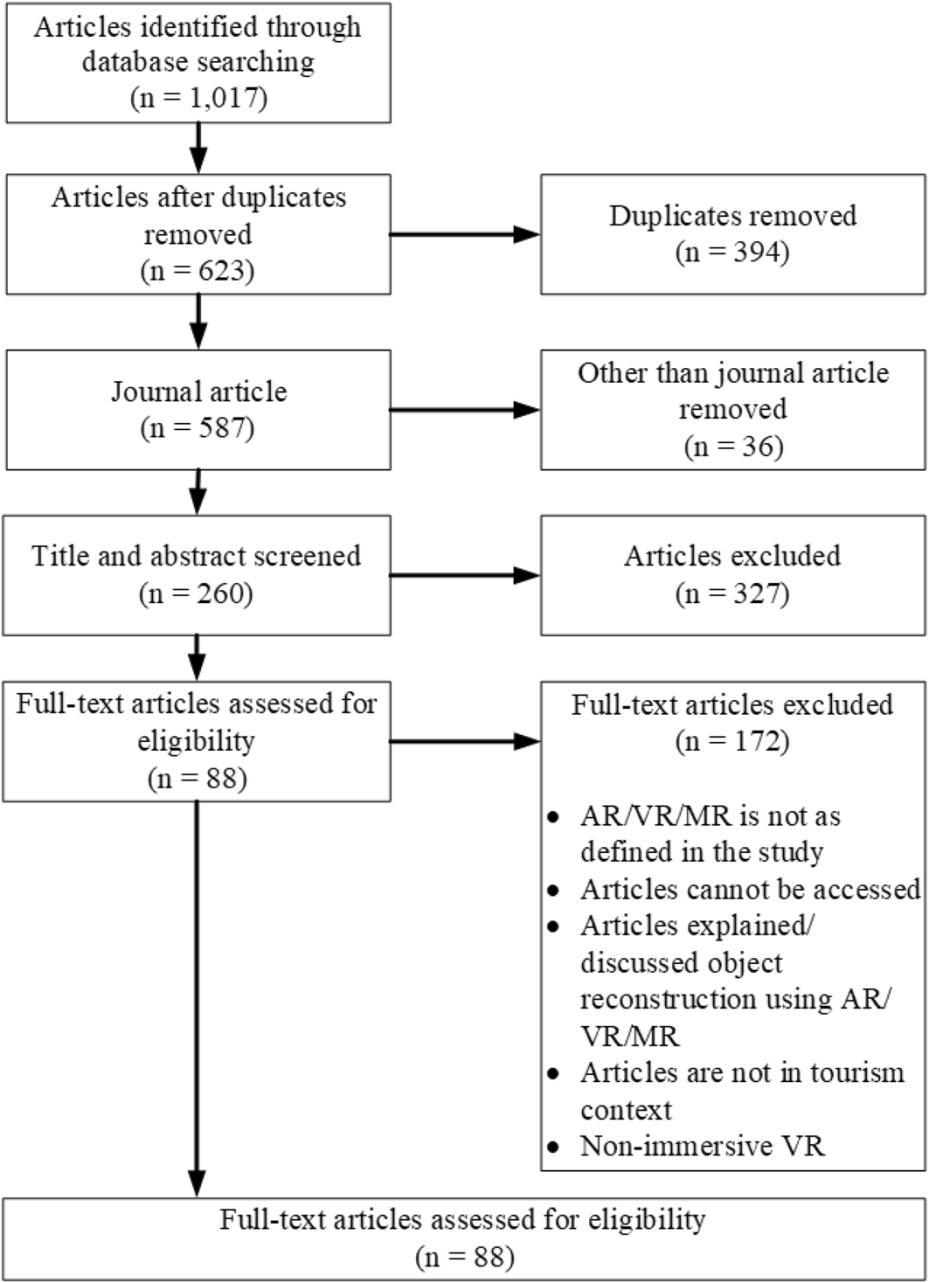
Article selection based on the PRISMA flow diagram

All articles identified in the search result were imported to the Endnote X9 bibliographic database (Clarivative Analytics 2019). The screening process followed the Preferred Reporting of Items for Systematic reviews and Meta-Analysis (PRISMA) flow diagram (Moher et al. 2009), as illustrated in Fig. 2. The articles were then subject to the three-level screening process.

The first level filtered studies to eliminate any (1) duplication, (2) anonymous studies, and (3) studies not published in a peer-reviewed journal as an original article. This reduced the number of articles from 1017 to 587.

In the second level, the titles and abstracts were sorted through to elucidate studies discussing AR, VR or MR in tourism. During this stage, 260 articles were deemed relevant to our study and then needed to be identified and assessed by reading the full text.

The third screening level involved full-text review to ensure that each article met the criteria, as listed in Table 3. This synthesis resulted in 88 relevant articles. The information from these articles was extracted and coded in Microsoft Excel before being reviewed and examined iteratively.

## Results and discussion

**Fig. 3:**
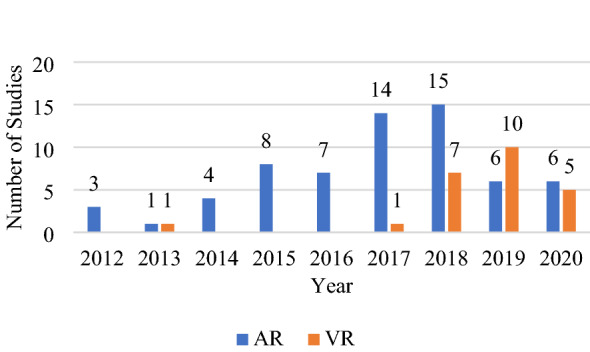
Article published distribution over time

This study aimed to illuminate some exciting aspects of immersive technology in tourism research. Immersive technology offers enormous potential in this domain. Given the specified inclusion and exclusion criteria, 88 peer-reviewed articles (see Appendix) published over the last nine years were relevant to this research topic. This review categorises the immersive technology from the selected articles into AR and VR based on the technology’s characteristics. Referring to Fig. 1, the technology used in several studies (Kasinathan et al. 2017; Nisi et al. 2018; Raptis et al. 2018; Hammady et al. 2020) might qualify as AR despite being referred to as MR. As seen in Fig. 3, AR has been a common immersive technology used in tourism research. In 2018, 15 articles on tourism research using AR were published, the highest number of articles to be published in the field in one year. In 2019, the number of articles on VR usage in tourism research peaked with ten articles published.

Table 5 shows the nature of the study in each of the selected articles. Design research and qualitative studies were dominant at 40.9%. A quantitative method, proceeded by experiences in immersive technology, was the most common data collection approach to capture participants’ experiences with and perceptions of the technology. The remaining articles were qualitative (8.0%), conceptual (5.7%) and mixed method (4.5%).

**Table 5:** Types of studies in the selected articles

Type of study	AR	VR	Total	(%)
Design research	32	4	36	40.9
Quantitative	17	19	36	40.9
Qualitative	6	1	7	8.0
Conceptual	5	–	5	5.7
Mixed method	4	–	4	4.5
Total	64	24	88	100.0

Table 6 focuses on the research locations of 47 empirical studies. Most research on immersive technology in tourism during the time defined in this study took place in Taiwan (14.9%), followed by the United Kingdom (12.8%) and the United States (10.6%). Four out of 47 empirical studies compared immersive technology usage in more than one country.

**Table 6:** Country distribution of 47 empirical articles based on the research location

Country	AR	VR	Total	(%)
*Single country*				
Taiwan	4	3	7	14.9
United Kingdom	4	2	6	12.8
United States	2	3	5	10.6
China	1	2	3	6.4
South Korea	3	–	3	6.4
Germany	1	1	2	4.3
Ireland	2	–	2	4.3
Italy	–	2	2	4.3
Portugal	2	–	2	4.3
England	1	–	1	2.1
Greece	1	–	1	2.1
Liechtenstein	1	–	1	2.1
Malaysia	1	–	1	2.1
Switzerland	–	1	1	2.1
Thailand	1	–	1	2.1
Not mentioned	1	4	5	10.6
*Multi-country*				
China & Taiwan	–	1	1	2.1
Hong Kong & United Kingdom	–	1	1	2.1
South Korea & Ireland	2	–	2	4.3
Total	27	20	47	100.0

Most of the articles listed in Appendix focused on tourism destinations and attractions, with few articles on immersive technology usage in tourism support such as hotel (Bogicevic et al. 2019; Israel et al. 2019; Zeng et al. 2020) and cruise ship (Yung et al. 2019) promotions. Some other tourism sectors, such as travel agencies (Bush 2022) and airlines (Emirates 2022), have been using VR to promote their products, but we did not find any articles within the selected literatures. A possible explanation is that some tourism sectors see the value of immersive technology, such as VR, as showing destination or location instead of the journey to the destination. Otherwise, there is still little or no research covering immersive technology usage in those tourism sectors.

We subjected the selected articles to the review process to better understand immersive technology in tourism and discover potential future research. The following sections elaborate on the selected articles' findings to answer the proposed research questions.

### The current state of immersive technology usage in tourism research (RQ1: What characteristics of immersive technology are used in tourism research?)

#### Augmented reality features in tourism research

Table 7 shows all the devices used in the selected AR-related articles. Mobile devices (smartphone or tablet PC) were the most common device used (76.3%). This is not surprising given that mobile devices are convenient to carry during travel and inexpensive compared to the other AR devices such as Microsoft HoloLens, Google Glass, or Meta One glasses.

**Table 7:** AR devices that were used in the empirical articles

Devices used in AR study	Number of studies	(%)
Mobile device	35	59.3
Wearable device	10	16.9
Other	2	3.4
Not mentioned	12	20.3
Total	59	100.0

AR combines a virtual object with the real environment in real time. The user can interact with the virtual object that blends the real world in three-dimensional perspectives (Azuma 1997). An AR system works in the presence of a trigger, which is a stimulus that initiates it to begin the virtual object augmentation on the device screen (Edwards-Stewart et al. 2016). Triggers can be a QR code printed on paper, an image, a real object, or a device location. Location-based AR was dominant in 37.3% of studies (see Table 8), while a trigger using a camera sensor, either markerless or marker-based, was present in 18.6% and 13.6% of studies, respectively. Four studies (6.8%) used AR with camera and location sensors as the trigger.

**Table 8:** Types of AR triggers used in the empirical articles

Trigger type	Number of studies	(%)
Location-based	22	37.3
Markerless	13	22.0
Marker-based	7	11.9
Spatial marking	4	6.8
Combination of marker-based and location-based	3	5.1
Combination of markerless and location-based	1	1.7
Not mentioned	9	15.3
Total	59	100.0

Some studies have built on the AR system’s capability to improve the user’s experience while exploring a location or object. Object recognition (markerless or marker-based) with geolocation feature addition is one example. The combined use of object recognition and geolocation provides spatial information for tour route decisions (Chu et al. 2012), improves the AR system’s accuracy, and makes it easier for the user to correctly recognise the object or place of interest and use that information in the future (Santos et al. 2017). Location-based AR uses a global positioning system (GPS) or beacon as the trigger. However, a beacon is preferable for indoor situations because building structures might block the signal used by GPS (Neumann et al. 1999). The combined AR trigger helps users explore a particular cultural site (Nisi et al. 2018; Gimeno et al. 2017) or city (Han et al. 2018; tom Dieck and Jung 2018).

The AR system’s integration with other technology is another option to enhance the user’s experience. This is more adaptive than a basic AR system and brings more relevant information to match users’ profiles and interests. Other people’s opinions also influence decision-making. For example, a person can obtain information from social media platforms such as Twitter about a tourism destination based on someone else’s opinion (Balduini et al. 2012, 2014). Social media might influence a person’s interest in visiting a tourism destination.

Several of the selected articles adopted cloud technology in the AR system. García-Crespo et al. (2016) proposed a framework for cultural entertainment centred on a smart city with AR that employs cloud-based technology. Moreover, two studies used cloud computing for media storage (Lee et al. 2017) and speech-based query processing (Lin and Chen 2017). Rodrigues et al. (2019) used an AR system that provides experiences through the five basic human senses. While the AR system delivers visual and audio representing two senses (sight and sound), the attached physical mobile device stimulates other senses: touch, smell and taste. It allows the user to have an immersive five-sense experience during object observation.

Spatial marking offers a different immersive level in AR. Four studies employed Microsoft HoloLens (Raptis et al. 2018; Hammady et al. 2020) and Meta One glasses (Pedersen et al. 2017; Oh et al. 2018). These devices take the immersion of AR a step further by overlaying digital objects without a trigger. Instead, the devices track through the user’s environment and anchor the digital object to the real environment on display. Little research exists in the tourism area regarding using these devices, and there are many related academic research opportunities.

#### Virtual reality features in tourism research

VR typically immerses the user in a computer-produced or alternative environment. The VR experience becomes realistic as the virtual environment blocks the user’s real-world view. Users immerse themselves in the experience and have a sense of belief that they appear in the alternate world through the help of devices such as head-mounted devices (HMDs) or ‘cave’-like rooms (Hobson and Williams 1995; Ghadban et al. 2013). An HMD unit is a device worn on the head, covering both eyes. HMDs can be low-cost and use a smartphone to show the virtual environment or more advanced, such as the Oculus Rift or HTC Vive. Alternatively, the user can experience VR in a room with a virtual environment projected onto all walls. When VR uses space in this way, it is called cave automatic virtual environment (CAVE).

As illustrated in Table 9, HMDs were the most popular devices (66.7%) in the reviewed articles. HMD is ideal for experiencing VR since the user’s view of the real-world is blocked entirely and replaced by a virtual environment. In some of the selected articles, VR was used to restore objects and the environment by generating a virtual environment to simulate a specific situation in the past (Kersten et al. 2018; Errichiello et al. 2019; Ghadban et al. 2013), for marketing (Lin et al. 2020), and for additional entertainment during visitation (Puig et al. 2020). Interestingly, more than half of the selected VR-related articles used VR with 360^O^ technology content (see Table 10). Although this meets VR’s characteristic of immersing the user in another world, it is not a computer-generated environment, and no user interactivity is involved. Instead of interacting with the virtual object, the user can only view the surrounding environment from a defined specific point of view. The 360° technology is a new form of photography and filmmaking recorded with a special camera. However, this has been widely known by most people as VR, due to the large amount of such content on YouTube and Facebook. Nonetheless, the 360° VR content might benefit market tourism destinations by simulating the real environment of a location. Hence, significant potential use of VR remains in certain aspects of tourism, such as planning and management, marketing, entertainment, education, accessibility, and heritage preservation (Guttentag 2010).

**Table 9:** VR devices used in the empirical articles

VR device	Number of studies	(%)
HMD	16	66.7
HMD and Computer	4	16.7
Computer	1	4.2
CAVE	1	4.2
Not mentioned	2	8.3
Total	24	100

**Table 10:** Types of VR content used in the empirical articles

VR content	Number of studies	(%)
360-video	8	33.3
Virtual environment	5	20.8
360-image	4	16.7
360 image and video	3	12.5
Virtual environment and 360-video	1	4.2
Not mentioned	3	12.5
Total	24	100.0

### Immersive technology applications within the tourism area (RQ2: To what extent does immersive technology play a role in the tourism visiting experience?)

**Fig. 4:**
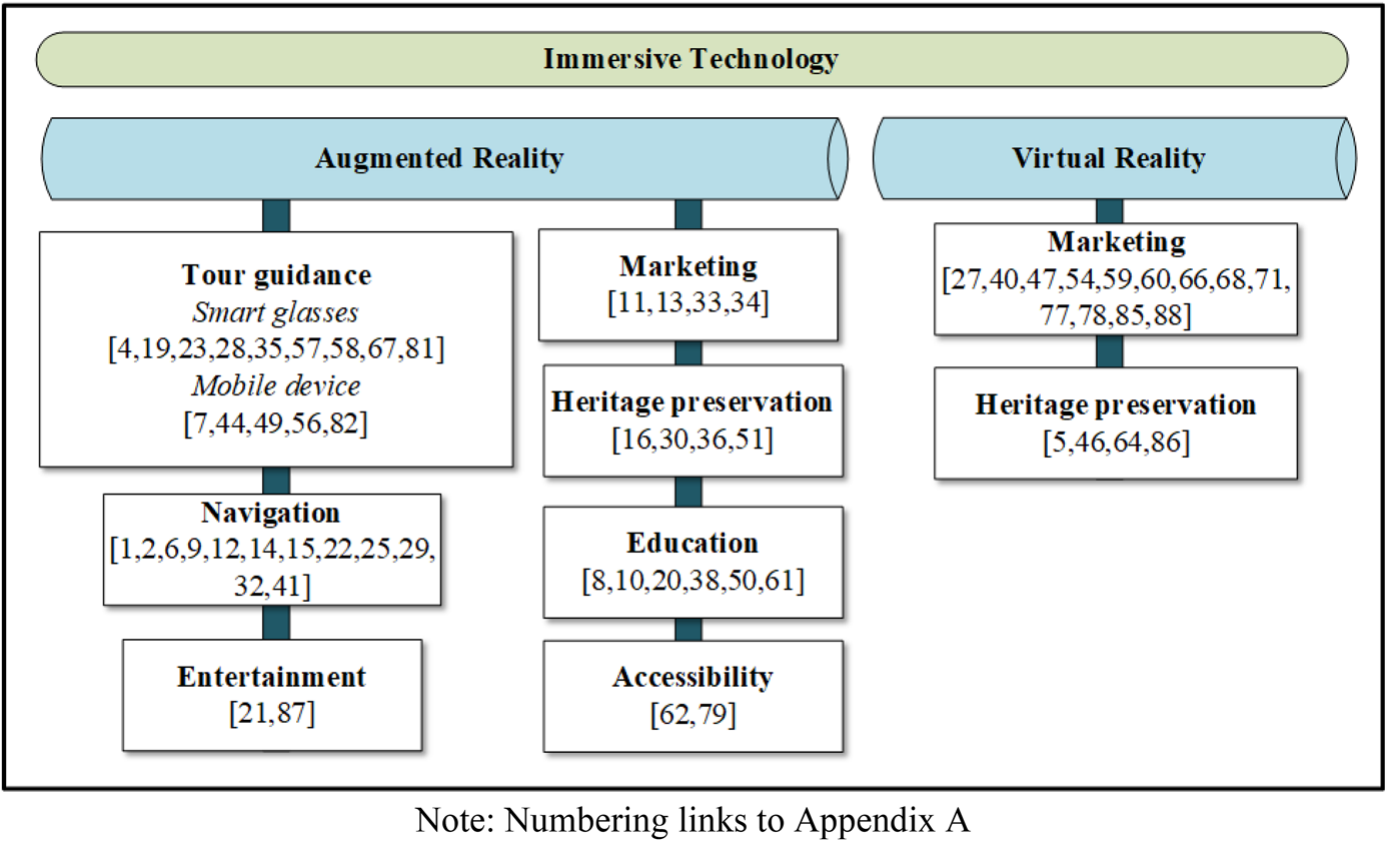
Immersive technology usage in tourism areas

Immersive technology offers academic and tourism stakeholders numerous opportunities in many tourism areas (see Fig. 4). Immersive technology usage has potential to improve tourism by increasing the number of visitors. It is also expected to increase awareness of lesser-known tourism destinations. This might be relevant because people are currently not travelling as much as before the global pandemic, and they might be interested in learning of new places. In this review, the tourism areas found in AR-related studies included AR for tour guidance, navigation, education, marketing, heritage preservation, entertainment, and accessibility. Previous studies also used VR for marketing and heritage preservation. The following section details the findings of each of the categories.

#### Immersive technology as a marketing tool

##### Augmented reality

Marketing is one of the tourism areas where immersive technology was implemented in the selected articles. The technology can serve as a promotional tool or facilitate research focusing on users’ intentions to visit the tourism destination. This review identified four studies that used AR as a promotional tool. Jung et al. (2015) observed the impact of marker-based AR system quality on the intention of visitors to Jeju Island to recommend others to use the AR system. They argued that the quality of the AR that covers the information content, system quality and service quality positively influences the user’s satisfaction, leading to the intention to recommend the AR system. This view is supported by Chung et al. (2015), who stated that the visual appeal of the AR system, with the support of adequate technical support, influences the user to use AR and visit the tourism destination. Other studies focused on how AR features promote tourism destinations, such as Lin and Chen (2017). They found that users engage more with the AR system if they feel that the videos of attractions that they post online can help other users. The next challenge is how the tourism provider persuades visitors to revisit the tourism destination. Lee et al. (2017) explored how mobile AR can increase tourists’ motivation to revisit a destination by exploring the post-travel experience using the entrance ticket as a scannable souvenir through the AR system.

##### Virtual reality

VR as a marketing tool in tourism research was more common than AR, specifically pre-visit tourism destination promotion. When potential tourists decide to visit a destination, they are likely to search for information about it or consider whether it is worth visiting. VR adoption in tourism creates opportunities to promote destinations (Cheeyong et al. 2017; Tussyadiah et al. 2018b; Adachi et al. 2020; Lin et al. 2020; Lin and Chen 2017). A qualitative study by tom Dieck et al. (2018c) reported that VR influences tourists to use the application, revisit the destination, recommend it to others and experience the destination from a different perspective (e.g. observing it from a helicopter instead of from the street). One of the characteristics of VR is a sense of presence. The users feel that their presence moves from the real world to the virtual world. VR provides a better sense of presence than AR, leading to increasing destination image formation (Yung et al. 2019), which leads to visit intention (Tussyadiah et al. 2018b). Experiencing VR with a HMD was also found to be a better promotion tool and provide better sensory stimulation and a more immersive experience compared to other systems (Flavián et al. 2019), such as a computer (Adachi et al. 2020), photographs (Yeh et al. 2017) or two-dimensional videos (Wagler and Hanus 2018). As a marketing tool, VR should provide content that represents the real conditions of the tourism destination. The tourism provider needs to ensure that the visual perspective of a destination they offer is genuine and as realistic as possible from the user’s perspective (Israel et al. 2019). However, the VR developer should consider the length of information if the content includes video (Marchiori et al. 2018). Additionally, in a recent quantitative study, Zeng et al. (2020) stressed that VR could add promotional value as an extension to online reviews.

#### Immersive technology for heritage preservation

One usage of AR and VR systems is reconstructing an object or environment since these systems produce computer-generated objects. AR systems enable the user to experience a three-dimensional virtual object based on the real heritage object, which might no longer exist in one piece or be possible to access. This way, the user can imagine and understand the object’s shape without looking at the real object.

##### Augmented reality

Four of the selected articles used AR for heritage preservation. Madsen and Madsen (2015) developed a three-dimensional visualisation of a castle chapel. The visitors experience the digital cultural heritage using a tablet connected to a large TV screen or a tablet PC. The authors argued that the AR system should provide more information and storytelling elements since the visitor only spends a short time using the AR system and does not fully explore the chapel. Another study by Gimeno et al. (2017) examined mobile AR for Casa Batlló, a landmark building in Spain. The AR system uses two approaches. First, it uses the gyroscope sensor and Bluetooth to trigger virtual objects to blend with the real world. As a result, the AR system augments the virtual modelled elements or furniture and blends this with the real world captured by the camera. Second, the user can scan the building’s physical model using the camera to see the virtual building on the screen, including detailed representations of the interior of each room on every floor of the building. Roongrungsi et al. (2017) designed a marker-based AR system to augment the Wat Phra Sri Rattana Mahathat temple. Panou et al. (2018) discussed the software architecture of an outdoor AR system that enables the user to experience virtual historical buildings around Chania, Greece. The system implements a gamification concept to let the user engage and interact more with cultural information.

##### Virtual reality

Other researchers have adopted VR to simulate heritage objects or buildings. A lab experiment by Ghadban et al. (2013) showed VR as an interactive environment to explore Hisham’s Palace in Palestine. The critical challenge of rebuilding the model was the remains of the physical building and the building’s limited history; both need to be right to ensure that the virtual, three-dimensional object is similar as possible to the real object in its time. Another example is a study by Kersten et al. (2018) that discussed a virtual model of a wooden model of Solomon’s temple at the Hamburg Museum using a VR system. The system enables the user to virtually experience the temple’s environment despite never visiting the temple in real life. Errichiello et al. (2019) observed the user experience in a past environment, particularly a ship launch during the Grand Tour of Naples and listening to music at San Teodoro Palace Hall Music. They argued that VR might be an effective way for visitors to enjoy a museum tour to obtain comprehensive information from different perspectives. The result showed that the users had a high intention of reusing the VR system and sharing their experience over the Internet. A mixed-method study by Puig et al. (2020) analysed the impact of a VR simulation of the Neolithic settlement of La Draga. The VR system provides a visual reconstruction of La Draga, where the user can interact with virtual Neolithic and non-Neolithic objects.

#### Immersive technology for education

This review categorises the usage of immersive technology to improve knowledge learning during visitation to a tourism destination. A crossover study by Sommerauer and Müller (2014) examined AR’s effect on gaining mathematical knowledge in an informal environment such as a museum. The authors concluded that AR could be a useful learning tool in formal and informal environments. A quasi-experimental study by Chang et al. (2015) observed mobile AR’s effectiveness in promoting learning performance at heritage sites in Taiwan. The authors stated that AR-guided participants acquired more knowledge about the heritage site than audio-guided and non-guided groups. Pendit et al. (2016) evaluated how AR might improve people’s enjoyment of learning about cultural heritage. The findings showed that the respondents enjoyed the AR’s cultural heritage learning experience. Tan and Lim (2017) implemented gamification in an AR system to improve visitors’ interest in exploring and learning about a historical place, Kellie’s Castle, in Malaysia. A study by Oh et al. (2018) used AR with Meta One glasses to observe how they can help users at a science museum learn about light refraction. The authors concluded that those who experienced game-based performance followed by non-game simulation performed better than a group who experienced these activities in the opposite order. A qualitative study by Yoon et al. (2018) observed an interactive AR used to learn about different types of scaffolds in a science museum.

#### Immersive technology as tour guidance

AR enhances the tourism experience in that the interactive virtual information overlays the real world. Our review found that tour guidance studies exclusively adopted AR technology, and it does appear to be the most appropriate technology to adopt when the user is physically located at the tourism destination. AR also provides additional interpretation resources to enhance user engagement with the observed object during visitation, significantly impacting the experience (Damala et al. 2013). The previous studies identified two types of devices for AR tour guidance: mobile devices and wearable devices (e.g. smart glasses).

##### Augmented reality with smart glasses

Smart glasses are wearable devices similar to regular eyeglasses equipped with a processing unit, various sensors and transparent lenses. The information displayed on the screen is integrated onto one or both lenses in front of the eyes, as if, from the AR user’s point of view, the digital information overlays the physical environment (Hein et al. 2017). Several studies employed wearable devices such as Google Glass (Mason 2016; tom Dieck et al. 2016; tom Dieck et al. 2018b; Tussyadiah et al. 2018a; Han et al. 2019a), HoloLens (Hammady et al. 2020) and Meta One (Pedersen et al. 2017). Using wearable devices reflects the relationship between the human body and technology, where the user senses the device as part of their body (Tussyadiah et al. 2018a). As a result, compared to an AR system that uses a mobile device, smart glasses offer a more immersive experience to the user, attractive and a balanced focus between the physical object and the device screen, while exploring tourism destinations (Mason 2016). Users were found to spend more time exploring the environment and engaging with the observed objects compared to without smart glasses (Hammady et al. 2020). On the other hand, tom Dieck et al. (2018b) found that some participants, on their first experience using smart glasses, tended to have a stronger recollection of the information provided by the device than the paintings because they tended to pay more attention to the device than the environment.

Some smart glasses have display limitations that might impact the displayed information. Participants in a study by Mason (2016) emphasised the difficulty in reading text on the Google Glass display due to length limitations. Hence, tom Dieck et al. (2016) stressed that the application content should provide detailed and suitable information to help users experience tourism. The information also needs to be delivered in real time to pique the user’s interest and allow an uninterrupted leisure experience (Han et al. 2019b; Choi and Kim 2017). Pedersen et al. (2017) supported the idea of implementing a reward system to lead users to more information and prompt them to proceed to the next object experience, thus making the visitation experience more enjoyable. Further, Damala et al. (2013) noted that the relevant content results from different stimuli induced during visitation rather than predefined content based on the user’s profile (e.g. adults, families).

##### Augmented reality using a mobile device

Modern mobile devices, such as smartphones or tablet PCs equipped with a camera, provide powerful computing to run AR-based applications. Because most mobile devices are less expensive than smart glasses, enhancing the tourism visitation experience is feasible. Given that so much information can be displayed on the device’s screen, observing how users divide their focus between the mobile device and the real object is interesting. A behavioural pattern study on painting appreciation by Chang et al. (2014) showed that users still enjoyed observing the real painting and did not look at the device’s screen excessively, although the AR system was considered a new technology for some of the study’s participants. Conversely, some participants in the Nisi et al. (2018) study reported feelings of isolation. The authors stated that the AR application indirectly made the users focus more on the smartphone screen than on physically interacting with the real object.

tom Dieck et al. (2018a) found that an AR system attached to a place encouraged visitors to engage more with the tourism destination. This view is supported by Nisi et al. (2018), who reported that the combination of storytelling and the observed physical environment stimulated users’ curiosity and willingness to explore that environment further, making the tourism experience educational and valuable. The information provided in the AR system is critical to providing a simple user interface with personalised information (Han et al. 2018) and interaction (tom Dieck and Jung 2018). Rather than shrinking an entire computer-based website layout to fit on a mobile device screen, the information must be adapted to suit a mobile layout (Chung et al. 2018). Interestingly, different cultural characteristics can result in different technological adaptations. According to Jung et al. (2018), people who live in cultures that prioritise the group over the individual and rely on social norms showed stronger dependence on social influence. Their decision to use tourism-based AR is likely based on the influence of friends and family.

#### Immersive technology as a navigation device

Some of the reviewed articles used immersive technology as a navigation device. We found that similar to the tour guidance applications, that navigation also exclusively relied on AR technology due to its connection to the physical realm. An AR system, such as those mainly used in smartphones, uses location sensors such as Bluetooth, GPS and compasses to pinpoint a specific location. Balduini et al. (2012) and Balduini et al. (2014) designed BOTTARI, an AR system that provides a point-of-interest recommendation in Seoul based on the social media community’s weighted opinions. The system continuously analyses social media streams and processes the information into personalised recommendations about places in the city. Chu et al. (2012) evaluated the Yehliu Geopark mGuiding system. The application implements AR using GPS coordinates from the mobile device. A study by Kourouthanassis et al. (2015b) examined eight mobile AR applications from prior studies to determine their design properties. A mobile AR application called CorfuAR implements Layar, an AR browser app, by following the design principles of the reviewed AR applications. The authors argued that the proposed design principles contributed to the mobile AR application’s high usability and performance, leading to better user–system interaction. A follow-up study by the same authors (Kourouthanassis et al. 2015a) confirmed that the functional properties of the application stimulate a feeling of pleasure, which leads to an increase in the intention to use the application. Siang et al. (2016) designed both the iMelaka 360 website and the iMelaka AR app to help tourists explore Melaka, Malaysia. Abidin et al. (2018) suggested an adaptive user interface for a location-based AR system to improve the tourist experience and ease access to Islamic tourism information, specifically in Malaysia.

#### Immersive technology adoption for other purposes

Another use of immersive technology in tourism was entertainment and accessibility support. A study by Shang et al. (2016) focused on using AR for post-visits. The mobile AR system used a postcard as a tourist souvenir to provide more information regarding the tourist destination that the user recently visited. Wu et al. (2020) investigated users’ behavioural intentions related to AR as part of the Avengers League World Tour exhibition in Taiwan. The users experienced the action from the point of view of the hero character.

Despite immersive technology offering many benefits to tourism, little research exists on immersive technology for disabled people. One design study by Baker et al. (2020) developed an AR tourism prototype for hard-of-hearing visitors. It is based on five conceptual elements: aesthetics, usability, interaction, motivation, and satisfaction. In a follow-up study, Baker et al. (2020) evaluated the prototype using groups of hard-of-hearing instructors, museum employees and experts. The prototype evaluation covered the interface, multimedia and interactivity.

### The potential challenge in using immersive technology in tourism (RQ3: what are the potential challenges of developing immersive technology for the tourism domain?)

**Fig. 5:**
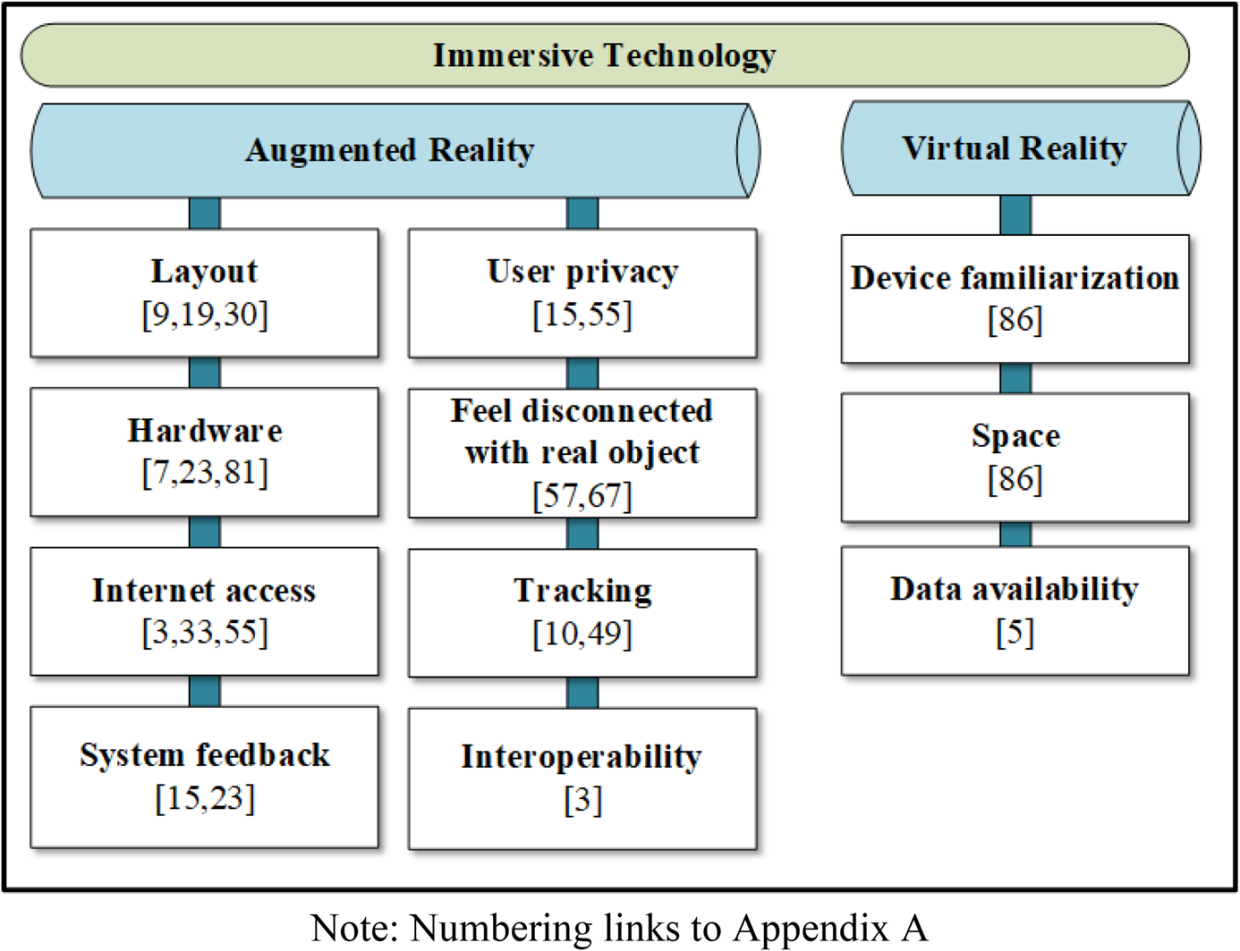
Challenges in immersive technology for tourism

While immersive technology shows significant potential use in tourism, it also has several challenges (see Fig. 5). This section discusses the challenges identified in the selected articles.

First, a lack of interoperability exists across device platforms (Kounavis et al. 2012). AR cannot be used across all the operating systems, albeit there are many frameworks and toolkits to develop the AR application. Second, some AR applications require an Internet connection to retrieve data from the server (Kasinathan et al. 2017). Some tourists consider mobile Internet expensive, and not all tourism areas or cities provide free Internet access (Kounavis et al. 2012; tom Dieck et al. 2018b). The third challenge lies in the physical size of the AR devices. Participants in a study by Chang et al. (2014) complained about the thick, heavy tablet PC used for painting appreciation. They indicated that a smaller device, like a smartphone, would be more suitable to carry as a tour guide device. In other studies, the drawbacks of tour guides using wearable devices were battery life (tom Dieck et al. 2016) and the device cost (Hammady et al. 2020).

The fourth challenge is the AR tracking ability when using a camera as a sensor. Camera-tracking AR, whether markerless or marker-based, should consider the amount of light and at what angle the camera faces the marker, picture or object. System responses, or feedback, are the fifth challenge of AR. The system should notify users of feedback errors to indicate the system’s process (Kourouthanassis et al. 2015b) and create personalised navigation (tom Dieck et al. 2016). The fifth challenge is feedback from the AR system. Real-time feedback from AR systems influenced user-system interaction. Users might experience a lower attitude toward using the system if they feel uncertain due to no response from the system (Kourouthanassis et al. 2015a). Participants in a study by tom Dieck et al. (2016) concerned about crashing and inadequate response from the system. System designers might need to minimize the possibility of system feedback issues to ensure users feel a smooth experience while using the AR system. The sixth challenge is the application layout. The layout of the annotation system influences the user’s perception of the observed area (Yovcheva et al. 2014). One participant in a study by Mason (2016) argued that it would be preferable for information to be shown via smart glasses rather than a mobile device screen. The seventh challenge identified in AR for tourism is the user’s engagement with the real object or surroundings. In an experimental design study by tom Dieck et al. (2018a), participants experienced a new AR technology that caused them to focus more on the device’s information than the paintings they were observing. This means that the application designer should ensure that the information projected at a specific time is not overloaded and thus does not distract from the leisure experience (Han et al. 2019a). Finally, user privacy is another concern regarding the use of AR in tourism. The benefit of content personalisation or a context-aware system delivers more related content to the user. However, if the system increasingly requests more personal details about the user, the risk of this data being lost or misused increases.

The challenges posed by VR in tourism are different from those posed by AR. The first challenge of using VR for tourism is device familiarisation. Puig et al. (2020) argued that familiarising the user with VR devices could be time-consuming. Further, the authors proposed combining the essentials of VR environment design with natural hand–gesture interaction that offers sufficient time flexibility to obtain information. The second challenge lies in the relationship between physical information from the real tourism destination and the virtual information in the VR environment. Puig et al. (2020) claimed that using information gained from the physical environment should help the user further explore information in a VR environment. Equally, the information from the virtual environment could help users learn about related tourism objects or situations. The third challenge of using VR for tourism is data availability. When presenting a virtual object, environment or scenario from the past, making the image presented in VR as realistic as possible relies on data availability.

## Conclusions and implications

This review builds on knowledge from existing reviews (Baker et al. 2017; Beck et al. 2019; Wei 2019; Yung and Khoo-Lattimore 2019). Findings from another study by Baker et al. (2017) revealed 11 major elements that need to be considered when designing mobile AR systems for hard-of-hearing individuals. Consideration of those elements could increase user engagement with AR applications in tourism. Findings from another VR-related review study (Beck et al. 2019) addressed that VR in tourism can be classified by its immersion level: non-immersive, semi-immersive or fully immersive. The major finding from Wei (2019) identified major constructs from prior studies and categorised them using the stimuli–dimensions–consequences framework. The framework synthesises key constructs associated with AR and VR in tourism and hospitality. Yung and Khoo-Lattimore (2019) explored AR and VR usage in the tourism sub-sector and revealed the methodology and theory implemented in prior studies on AR and VR in tourism. Based on the existing review findings, the present study’s findings extend the knowledge on AR/VR usage in tourism. We have identified AR and VR as the immersive technology used in the selected research articles.

The following section elaborates on the potential future research on immersive technology in tourism and recommendations for stakeholders. This section also includes identified limitations of this study that might need improvement in future studies.

### Limitations of the study

Although this review provides detail on immersive technology research in tourism, some limitations would be helpful to consider during future research. First, we found that some articles related to tourist attractions such as cultural heritage and museums did not appear in the search results. Future research might include specific tourism attractions as keywords in the search query. Second, our inclusion was limited to peer-reviewed journal articles. Our findings indicate increasing immersive technology adoption in tourism-related articles. Based on that trend, it could help to expand the findings by including conference proceedings that, to avoid duplication, are not extended to journal articles. Third, immersive technology in tourism indicates an increased number of published articles in line with time. However, the lack of technology adoption by the tourism providers either due to cost or lack of understanding remains unclear. Further study might focus on the challenge of adopting the technology.

Finally, the oldest article included in this review was published in 2012. Current technology has made substantial advances since then, and the potential challenges in technology adoption in tourism might have evolved too over time. For example, AR technology is more mature, with state-of-the-art mobile devices and AR integration with light detection and ranging (LiDAR). Recent VR technology can also deliver high-quality images with recent computation. For further reviews, we suggest that this may be a justification to adopt shorter review windows, for example of 5 years.

### Future works

#### Integrating immersive technology with other technology to enhance the user experience

This review identified the types of immersive technology used in tourism articles. We observed that only AR and VR appeared in prior studies. Therefore, several potential directions for future research could implement another type of immersive technology under the MR umbrella and another technology integration. AR systems are used dominantly in mobile devices using a trigger to initiate the digital content on the screen, overlaying the real-world view. Modern smart devices are powered with high system specifications that quickly load the AR application. One direction for future research could be to use AR with LiDAR to detect the user’s environment. Using that technique, AR could help promote the tourism destination (Lee et al. 2017; Lin and Chen 2017) or enhance the user experience during visitation (Rodrigues et al. 2019; Yoon et al. 2018; Nisi et al. 2018). Likewise, another direction for future research with AR could be to use a wearable device to measure visitor responses to an enhanced experience during visitation (Hammady et al. 2020; Han et al. 2019a; Tussyadiah et al. 2018a). Although AR with wearable devices such as Google Glass and HoloLens is still considered expensive, its usage can deliver a seamless experience without requiring the user to hold the device. A third direction for future research could be to assess visitor responses on a multi-trigger AR system to improve the destination exploration experience using marker and location sensors.

Traditionally, VR visualises a virtual environment fully generated by a computer. The popularity of 360° technology in line with various HMD availability opens the opportunity for tourism providers to create a VR experience using a 360° camera without high-level programming knowledge. The following research agenda could be used to investigate the difference between using a computer modelling VR content and a 360° image or video for different situations, such as pre-visit or promotion, during visitation and post-visitation.

#### Immersive technology applications within the tourism area

Immersive technology has various uses in tourism. We found that AR is used primarily for tour guidance and navigation, and VR is mainly used to promote tourism destinations. One possible future research direction is to examine AR usage, especially personalisation based on visitor age, to enhance the learning experience during visitation (tom Dieck et al. 2018b; Yoon et al. 2018).

A potential direction for future VR-related research is to assess whether the developed application reflects the expected specific environment, such as VR content that gives the user the sensation that they are experiencing a situation in the past (Puig et al. 2020; Errichiello et al. 2019). Another potential research agenda focuses on cultural heritage since VR can preserve heritage objects or situations and represent them using digital objects. It could also be interesting to explore immersive technology in areas other than those identified in this study, such as VR applications to support accessibility for disabled people and its potential to replace actual visitation due to physical restrictions.

#### Potential challenge in using immersive technology in tourism

The selected articles indicate several potential challenges of using immersive technology in tourism. They can give tourism stakeholders, primarily application developers, insight into designing a suitable system to meet users’ needs. Some challenges can be solved using current technology. For example, the interoperability issue (Kounavis et al. 2012) can be solved by developing the AR application using Unity (Unity Technologies 2020). Tracking issues that occur while detecting markers (Nisi et al. 2018) can be handled by using smartphones with an up-to-date camera sensor and using a new technique for spatial markings, such as LiDAR. Another challenge we found is that users feel disconnected from the real object while using the AR application. Application developers must consider the balance of interactivity between exploring the actual object and using the application. Tourism providers can also support the user’s experience by designing an interactive and attractive display presentation. Future research might focus on the design aspect of immersive technology for tourism and its evaluation. Exploring the impact of content-aware immersive technology on providing information based on the user’s characteristics would also be interesting.

### Recommendations for stakeholders

#### Recommendations for the system developer

Our research shows that most AR applications use one trigger type to initiate the virtual object. As the user moves around the destination, the application is expected to recognise the user’s preference and recommend the next object that they need to explore. This can be achieved using traditional triggers such as a camera and a location sensor to detect the user’s position. In addition, the user experience can be assessed to improve the application and learn visitor preference. We also found that the visitor may engage with the AR application more than the real object or environment itself. Therefore, the AR application developer might consider designing an interactive application that will let the visitor examine the real object with additional information from the application.

#### Recommendations for tourism providers

Our research shows that AR is mainly used during actual visitation at the tourism destination. AR can enhance the user experience while the user is exploring the destination. Therefore, it might be helpful for the tourism provider to consider the layout of the destination to ensure that it supports the AR application usage. Infrastructure such as Internet connection, room layout, and booth layout can be developed to achieve this. Users might immerse themselves in the environment with the addition of AR applications and thus focus not only focus on the virtual object that appears on the device screen but also on any objects in the real environment.

To reiterate, we found that VR is mainly used to promote tourism. Undoubtedly, VR is gaining recognition as a solution simulating a realistic environment. Thus, our recommendation for tourism providers is to introduce the destination via VR through a travel agent (Bush 2022), meaning that potential visitors can experience the destination before deciding on travel. An alternative is to integrate VR with the destination’s website to help website users gain more information regarding the tourism destination. Another recommendation is to integrate VR with other applications, such as the Conqueror (Home Run Limited 2021), a virtual travel application that gained popularity during travel restrictions due to the COVID-19 global pandemic. The application provides many virtual challenges to complete at well-known destinations worldwide. When users join a challenge, they can gain the distance they achieved through their daily exercise such as running, walking or cycling, which translates to distance travelled. The tracked distance can be synchronised with the Conqueror application to travel virtually to the selected destination challenge. Users can explore the route along the virtual trip. Tourism providers can integrate VR about their destinations with the application to enable users to have a VR experience of the promoted destination.

Tourism providers should consider VR adoption since it brings benefits as a virtual tour for users and them. Users may use virtual tours for cost-effectiveness, health safety and time-saving. Specific users such as the elderly or those with physical disabilities would feel safer, secure, and require no special equipment to enjoy the virtual trip (Scott 2020). As for tourism providers, VR adoption creates employment opportunities for content creators, videographers and tour guides (Scott 2020). Further, VR can be programable (Sussmann and Vanhegan 2000) to keep the content and information up-to-date.

## Conclusion

This review explored the use of immersive technology in the context of tourism through a comprehensive review of 88 articles published between 2012 and 2020. The increasing number of journal articles published in this field reflects the research interest in immersive technology for tourism, primarily in AR. This work advances prior works and reviews through several contributions. We have identified AR and VR combined with other technology can offer potential user experience enhancement. We have also identified immersive technology usage within the tourism sub-sector and potential challenges of using immersive technologies. This review paper generates an overview that both academic and tourism stakeholders can use to understand better the current progress and possible research directions on immersive technology in tourism. Immersive technology, such as AR and VR, has numerous real-world applications and the potential to spark new interest and uptake of travel destinations which have been lagging in recent years. It is hoped that this review stimulates further research both in applying this technology to novel contexts and taking advantage of cutting-edge VR technology which has become increasingly available in the consumer space.

## Data Availability

We do not analyse or generate any datasets, because our work proceeds within a theoretical approach.

## Appendix: Full list summary of 88 selected articles

**Table:**

Nos.	Authors	Research location	AR/VR	Type of work	Focused tourism place/object	Technology used
1	Balduini et al. (2012)	South Korea	AR	Design research	City’s point of interest	Location-based; BOTTARI app; Smartphone /Tablet; Twitter
2	Chu et al. (2012)	China	AR	Design research	Park	Location-based; Geopark app; Smartphone
3	Kounavis et al. (2012)	Not available	AR	Conceptual	Not mentioned	Not mentioned
4	Damala et al. (2013)	Not available	AR	Conceptual	Museum	Eye-tracking; Gesture-tracking; Audio-tracking; Physiological sensing; ARtSENSE; Smartglasses
5	Ghadban et al. (2013)	Palestine	VR	Design research	Cultural heritage	Virtual environment; U-shape theatre projector; Polarized-glasess; Controller
6	Balduini et al. (2014)	South Korea	AR	Design research	City’s point of interest	Location-based; BOTTARI app; Smartphone/Tablet; Twitter
7	Chang et al. (2014)	Taiwan	AR	Empirical—mixed method	Museum	Markerless-based; Tablet PC
8	Sommerauer and Müller (2014)	Liechtenstein	AR	Empirical—quantitative	Exhibition	Marker-based; Smartphone; Aurasma Glued
9	Yovcheva et al. (2014)	United Kingdom	AR	Design research	City’s point of interest	Location-based; Smartphone; Junaio; LocalScope; Wikitude; AccrossAir
10	Chang et al. (2015)	Taiwan	AR	Empirical—mixed method	Cultural site	Markerless-based; Tablet
11	Chung et al. (2015)	South Korea	AR	Empirical—quantitative	Palace	Location-based; Deoksugung In My Hand app; Smartphone
12	Grubert et al. (2015)	Austria	AR	Design research	Ski resort	Markerless-based; Smartphone
13	Jung et al. (2015)	South Korea	AR	Empirical—Quantitative	Theme park	Marker-based
14	Kourouthanassis et al. (2015a)	Greece	AR	Design research	City’s point of interest	Location-based; CorfuAR app; Layar
15	Kourouthanassis et al. (2015b)	Greece	AR	Design research	City’s point of interest	Location-based; CorfuAR app; Layar
16	Madsen and Madsen (2015)	Denmark	AR	Design research	Museum	Markerless-based; Tablet
17	Damala et al. (2016)	Netherlands	AR	Design research	Museum	Markerless-based; Smartphone
18	García-Crespo et al. (2016)	Not available	AR	Design research	City	Location-based; Cloud-based integration
19	Mason (2016)	United States of America	AR	Design research	Museum	Marker-based; Google Glass
20	Pendit et al. (2016)	Malaysia	AR	Design research	Cultural site	Location-based; AR@Melaka
21	Shang et al. (2016)	Malaysia	AR	Design research	Not mentioned	Markerless-based
22	Siang et al. (2016)	Malaysia	AR	Empirical—quantitative	City	Location-based;
23	tom Dieck et al. (2016)	England	AR	Design research	Art gallery	Markerless-based; Museum Zoom app; Google Glass
24	Trojan (2016)	Czech	AR	Design research	City’s point of interest	Integrating AR services for the masses: geotagged POI transformation platform
25	Aluri (2017)	United States of America	AR	Empirical—quantitative	Not mentioned	Location-based; Pokemon Go; Smartphone
26	Bogomazova and Stenyushkina (2017)	Russia	AR	Conceptual	Not mentioned	Not mentioned
27	Cheeyong et al. (2017)	South Korea	VR	Design research	Marine leisure sport	360-video; Head Mounted Display
28	Choi and Kim (2017)	South Korea	AR	Design research	Museum	Location-based; Head Mounted Display
29	Cushing and Cowan (2017)	Ireland	AR	Design research	Cultural site	Location-based; Walk1916 app; Smartphone
30	Gimeno et al. (2017)	Spain	AR	Design research	Museum	Marker-based; Location-based; Smartphone
31	Jung and tom Dieck (2017)	United Kingdom	AR	Conceptual	Museum	Marker-based
32	Kasinathan et al. (2017)	Not available	AR	Design research	Not mentioned	Location-based
33	Lee et al. (2017)	Taiwan	AR	Empirical—quantitative	Cultural site	Markerless-based; Smartphone
34	Lin and Chen (2017)	Thailand	AR	Empirical—quantitative	Ethnic	Markerless-based; Smartphone; Google Speech API; Samsung text-to-speech engine
35	Pedersen et al. (2017)	Canada	AR	Design research	Museum	Tombseer app; Meta Developer Kit
36	Roongrungsi et al. (2017)	Thailand	AR	Design research	Cultural site	Marker-based; ARCH-TOUR
37	Shukri et al. (2017)	Not available	AR	Conceptual	Not mentioned	Not mentioned
38	Tan and Lim (2017)	Malaysia	AR	Design research	Cultural site	Markerless-based; MIGHT
39	tom Dieck and Jung (2017)	United Kingdom	AR	Empirical—qualitative	Museum	Not mentioned
40	Yeh et al. (2017)	Taiwan	VR	Empirical—quantitative	City’s point of interest	360-image; Computer
41	Abidin et al. (2018)	Not available	AR	Design research	Not mentioned	Location-based
42	Chung et al. (2018)	South Korea	AR	Empirical—quantitative	Palace	Location-based; Deoksugung In My Hand app; Smartphone
43	Fenu and Pittarello (2018)	Italy	AR	Design research	Museum	Markerless-based; Smartphone; Wikitude
44	Han et al. (2018)	Ireland	AR	Empirical—qualitative	City’s point of interest	Marker-based; Location-based; DublinAR app; Smartphone
45	Jung et al. (2018)	South Korea; Ireland	AR	Empirical—quantitative	Palace, Post office	Marker-based; Location-based; DublinAR app; Deoksugung In My Hand app; Smartphone
46	Kersten et al. (2018)	Germany	VR	Design research	Museum	3D-modelling; HTC Vive
47	Marasco et al. (2018)	Italy	VR	Empirical—quantitative	Cultural site	360-video; Oculus Rift
48	Marchiori et al. (2018)	Switzerland	VR	Empirical—quantitative	City’s point of interest	360-image; 360-video; Oculus Rift
49	Nisi et al. (2018)	Portugal	AR	Design research	Museum	Marker-based; Yasmin Adventure app; Smartphone
50	Oh et al. (2018)	South Korea	AR	Design research	Museum	ARfract app; Meta One
51	Panou et al. (2018)	Greece	AR	Design research	Cultural site	Location-based; Smartphone
52	Paulo et al. (2018)	Portugal	AR	Empirical—quantitative	Not mentioned	Mobile device
53	Raptis et al. (2018)	Greece	AR	Empirical—mixed method	Cultural site	Holo Tour; HoloLens; Tobii Pro Glasses 2
54	tom Dieck et al. (2018c)	United Kingdom	VR	Empirical—qualitative	Park	360-video; Google Cardboard
55	tom Dieck and Jung (2018)	Ireland	AR	Empirical—qualitative	City’s point of interest	Marker-based; Location-based; DublinAR app; Smartphone
56	tom Dieck et al. (2018a)	England	AR	Empirical—quantitative	City’s point of interest	Location-based; iBeacon; Smartphone
57	tom Dieck et al. (2018b)	United Kingdom	AR	Empirical—qualitative	Art gallery	Museum Zoom app; Google Glass
58	Tussyadiah et al. (2018a)	United Kingdom	AR	Empirical—quantitative	Art gallery	Museum Zoom app; Google Glass
59	Tussyadiah et al. (2018b)	Hongkong; United Kingdom	VR	Empirical—quantitative	City; Park	360-image; 360-video; Google Cardboard; Samsung Gear VR
60	Wagler and Hanus (2018)	United States of America	VR	Empirical—quantitative	Capitol building	360-video; Oculus Rift
61	Yoon et al. (2018)	United States of America	AR	Empirical—Mixed method	Museum	Interactive device
62	Baker et al. (2019)	Iraq	AR	Design research	Museum	Marker-based
63	Bogicevic et al. (2019)	United States of America	VR	Empirical—quantitative	Hotel	360-image; HTC Vive; Laptop
64	Errichiello et al. (2019)	Italy	VR	Empirical—quantitative	Cultural site	3D modelling; Samsung Oculus Gear
65	Fang and Lin (2019)	Taiwan	VR	Empirical—quantitative	City	360-image; 360-video; Google Street; Veer VR; VR Box
66	Flavián et al. (2019)	Not available	VR	Empirical—quantitative	City; Canyon	360-video; HMD; Computer
67	Han et al. (2019a)	United Kingdom	AR	Empirical—qualitative	Art gallery	Google Glass
68	Israel et al. (2019)	Germany	VR	Empirical—quantitative	Hotel	360-image; Gear VR
69	Kassim et al. (2019)	Not available	AR	Design research	Museum	Not mentioned
70	Koo et al. (2019)	South Korea	AR	Design research	Fortress	Markerless-based; Smartphone
71	Lee et al. (2019)	United Kingdom	VR	Empirical—quantitative	Museum	360-video; Samsung Gear VR
72	Li and Chen (2019)	China	VR	Empirical—quantitative	Cultural site	HMD
73	Rodrigues et al. (2019)	Portugal	AR	Empirical—quantitative	Museum	Location-based; Portable device for touch, taste and smell (PDTTS); Smartphone
74	Tsai (2019)	China	AR	Empirical—quantitative	Cultural site	Location-based
75	Wei et al. (2019)	United States of America	VR	Empirical—quantitative	Theme park	Not mentioned
76	Wu et al. (2019)	Taiwan	VR	Empirical—quantitative	City	360-video; Oculus
77	Yung, Khoo-Lattimore, and Potter (2019)	Not available	VR	Empirical—quantitative	Cruise ship	360-video; Princess Cruise; HTC Vive
78	Adachi et al. (2020)	Not available	VR	Empirical—quantitative	City’s point of interest	360-video; Samsung Gear VR; Computer
79	Baker et al. (2020)	Not available	AR	Design research	Museum	Marker-based; Smartphone
80	Cranmer et al. (2020)	Germany	AR	Empirical—qualitative	Tourism trade fair	Not mentioned
81	Hammady et al. (2020)	Egypt	AR	Design research	Museum	HoloLens
82	Jung et al. (2020)	South Korea; Ireland	AR	Empirical—quantitative	Palace; Museum	Markerless-based; DublinAR app; Deoksugung In My Hand app
83	Kim et al. (2020)	Not available	VR	Empirical—quantitative	Not mentioned	Not mentioned
84	Lacka (2020)	Not available	AR	Empirical—quantitative	Not mentioned	Location-based
85	Lin et al. (2020)	China; Taiwan	VR	Empirical—quantitative	Painting	3D modelling; HTC Vive
86	Puig et al. (2020)	Spain	VR	Design research	Cultural site	3D modelling; Draga 360; HTC Vive
87	Wu et al. (2020)	Taiwan	AR	Empirical—quantitative	Exhibition	Not mentioned
88	Zeng et al. (2020)	China	VR	Empirical—quantitative	Hotel	360-image; HMD
